# Supplementary material for: Scanning nuclear resonance imaging of a hyperfine-coupled quantum Hall system
Source: Nat Commun. 2018 Jun 7;9:2215. doi: 10.1038/s41467-018-04612-y (PMC5992213; doi:10.1038/s41467-018-04612-y)
Supplement: Supplementary file 1 — Supplementary Information [file 41467_2018_4612_MOESM1_ESM.pdf]

Supplementary Information

Hashimoto, K. *et al.*

Scanning nuclear resonance imaging of a hyperfine-coupled quantum Hall system.

Supplementary Note 1. Electric quadruple transition

The total spin $I = 1/2$ has only the magnetic dipole moments used in the standard nuclear magnetic resonance; however, quadrupolar nuclear spins such as the total spin $I = 3/2$ for ^{75}As, ^{69}Ga, and ^{71}Ga nuclei additionally have the electric quadrupole moments comprising non-spherical positive charge distribution. The electric moments of nuclear spins interact with the E-field gradient through the electric quadrupole interaction[1, 2]:

$$H_\mathrm{Q} = \sum_{m=-2}^{2} \boldsymbol{Q}^m \boldsymbol{V}^{-m}, \tag{1}$$

where $\boldsymbol{Q}^m$ is the nuclear quadrupole operator and $\boldsymbol{V}^{-m}$ is the electric field gradient operator. By defining the dimensionless nuclear spin angular momentum operators (I) and its components of, I_x, I_y, and I_z and $I_\pm = I_x \pm iI_y$ under a static magnetic field applied in the z direction, the components of $\boldsymbol{Q}^m$ can be written as

$$\begin{aligned} Q^0 &= \frac{1}{2}A[3I_z^2 - I(I+1)] \\ Q^{\pm 1} &= \frac{\sqrt{6}}{4}A[I_z I_\pm + I_\pm I_z] \\ Q^{\pm 2} &= \frac{\sqrt{6}}{4}A(I_\pm)^2, \end{aligned} \tag{2}$$

where $A = eQ/[I(2I-1)]$ with the electric quadrupole moment Q. The component of $\boldsymbol{V}^m$ in Supplementary Eq. 1 can be written as

$$\begin{aligned} V^0 &= -\frac{1}{2}V_{zz}, \\ V^{\pm 1} &= \mp\frac{\sqrt{6}}{6}(V_{xz} \pm iV_{yz}), \\ V^{\pm 2} &= \frac{\sqrt{6}}{12}(V_{xx} - V_{yy} \pm 2iV_{xy}). \end{aligned} \tag{3}$$

When the external electric field E_k is applied, the E-field gradient tensor V_{ij} can be expanded as

$$V_{ij} = (V_{ij})_{E=0} + C_{ij,k}E_k + \cdots, \tag{4}$$

where $C_{ij,k} = (\delta V_{ij}/\delta E_k)_{E=0}$, and i, j, and k are a permutation of the three crystalline axes [100], [010], and [001] denoted as x, y, z. Considering the cubic/tetrahedral symmetry in GaAs, $(V_{ij})_{E=0} = 0$ and $C_{ii} = 0$, so that only nonzero components of $C_{ij,k}$ is given at $i \neq j \neq k$. Hence, Supplementary Eq. 4 is reduced[3, 4] to

$$V_{ij} = V_{ji} = C_{ij,k}E_k, \tag{5}$$

where

$$C_{ij,k} = \begin{array}{c} \\ xx \\ yy \\ zz \\ xy \\ xz \\ yz \end{array} \begin{array}{c} \begin{array}{ccc} E_x & E_y & E_z \end{array} \\ \begin{pmatrix} 0 & 0 & 0 \\ 0 & 0 & 0 \\ 0 & 0 & 0 \\ 0 & 0 & 1 \\ 0 & 1 & 0 \\ 1 & 0 & 0 \end{pmatrix} \end{array} \beta, \tag{6}$$

and β is a constant dependent on a nuclide. By substituting Supplementary Eq. 5 to Supplementary Eq. 3, the component of $\boldsymbol{V}^m$ reads

$$\begin{aligned} V^0 &= 0, \\ V^{\pm 1} &= -\frac{\sqrt{6}}{6} i C_{12,3}(E_1 \mp i E_2), \\ V^{\pm 2} &= \pm \frac{\sqrt{6}}{6} i C_{12,3} E_3, \end{aligned} \tag{7}$$

where x, y, z are denoted as 1, 2, 3, respectively. Eventually, the $\Delta m = \pm 1$ and ± 2 transition probability based on electric quadrupole interaction are

$$\begin{aligned} W_{m,m\pm 1} &= \frac{1}{4\hbar^2} \langle m|H_Q|m \pm 1\rangle^2 g_1(f) \propto |V^{\pm 1}|^2 = \frac{C_{12,3}^2}{6}(E_1^2 + E_2^2), \\ W_{m,m\pm 2} &= \frac{1}{4\hbar^2} \langle m|H_Q|m \pm 2\rangle^2 g_2(f) \propto |V^{\pm 2}|^2 = \frac{C_{12,3}^2}{6} E_3^2, \end{aligned} \tag{8}$$

where $g_1(f)$ and $g_2(f)$ are the absorption line shape factors for the $\Delta m = \pm 1$ and ± 2 transitions (see Fig. 1b), respectively. Thus, the $\Delta m = \pm 1$ and ± 2 NR can be driven by the RF E field at the fundamental NR frequency and twice the NR frequency. In this study, the z ([001]) component of the RF E field normal to the 2D-electron gas plane dominantly drives the $\Delta m = \pm 2$ transitions.

Supplementary Note 2. Compensation for potential mismatch between tip and sample

To minimise the electrostatic influence of the tip, we examined tip-induced influence on V_x at different DC bias voltages of the tip (V_{tip}). Supplementary Figure 1a shows the V_x line profiles obtained by scanning the DC-biased tip across the Hall bar at a magnetic field $B = 0$ T. To compare the tip-induced influence on the transport within the Hall bar, the line profiles were plotted after subtracting the value of V_x obtained outside the Hall bar. A negative (positive) increase in V_{tip}

decreased (increased) the electron density of the two–dimensional electron gas, and therefore, it led to a rise (drop) in V_x in the Hall bar (position: approximately 7–17 μm). The influence of the tip was almost completely suppressed at $V_{\rm tip} = 0.2$ V, indicating that the potential mismatch, for example, due to the workfunction mismatch, was compensated for. Thus, we performed NR measurements at $V_{\rm tip} = 0.2$ V.

Further, the imposed source–drain voltage could be an additional source of local potential mismatch between the tip and sample surface. We tested the influence of the potential mismatch on an edge pattern such as the one shown in Fig. 2d by changing $V_{\rm tip}$ instead of the source-drain voltage. Supplementary Figure 1b shows the line mappings of the nuclear resonance intensity captured at different $V_{\rm tip}$ along the same line across a Hall bar. The peak pattern near $y = 2.5$ μm persists in the $V_{\rm tip}$ range of ± 0.4 V with respect to $V_{\rm tip} = 0.2$ V used for compensating the work function difference such that the corresponding range of the potential mismatch does not affect the resistive detection sensitivity. We thus concluded that the source-drain voltage used in our measurements $V_{\rm sd} < 40$ mV did not affect the detection sensitivity.

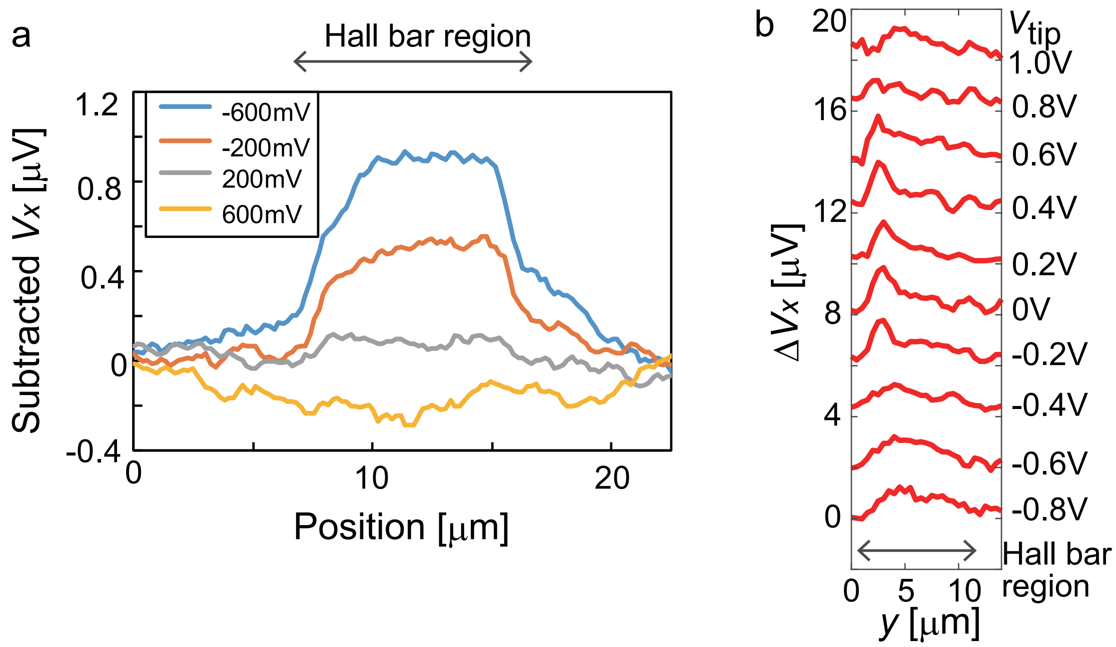

Supplementary Figure 1: **DC tip voltage dependence of the line profile across the Hall bar. a.** Line profiles of V_x recorded at $B = 0$ T, $I_x = 2$ μA, and electron density $n_{\rm s} = 1.7 \times 10^{15}$ m^{-2}. The $V_{\rm tip}$ are indicated in the figure. The profile of each line was obtained by spatially averaging 48 lines over the 9.5–μm region. **b.** Line profiles of the NR intensity across the Hall bar at $\nu = 1.05$, $I_x = 0.6\mu$A, and $B = 8$ T. The NR intensity was obtained by recording V_x reduction, ΔV_x, due to the NR resulting from E-field application at an on-resonance frequency of 115.75 MHz for 6 s. The curves are vertically offset for clarity.

Supplementary Note 3. Dynamic nuclear polarization and its resistive detection

For the 2D electron gas in the GaAs quantum well, an electron spin $\boldsymbol{S}$ within the s-type conduction bands interacts with a nuclear spin $\boldsymbol{I}$ through the Fermi contact hyperfine Hamiltonian:

$$H_{\mathrm{HF}} \propto A_{\mathrm{H}} \boldsymbol{I} \cdot \boldsymbol{S} = \frac{A_{\mathrm{H}}}{2}(I^{+}S^{-} + I^{-}S^{+}) + A_{\mathrm{H}} I_{\mathrm{z}} S_{\mathrm{z}}, \quad (9)$$

where A_{H} is the hyperfine coupling constant. The first term represents the simultaneous process of the electron spin flip and nuclear spin flop. This flip-flop process is accommodated with the inter Landau level scattering between LL0↑ and LL0↓ in the QH breakdown regime. It transfers spin angular momentum to a nuclear spin subsystem, resulting in current-induced dynamic nuclear polarization.

The second term describes static interaction between the z components of the electron (S_z) and the nuclear (I_z) spin. When the nuclear spins are polarised $\langle I_z \rangle$, the surrounding electrons experience an additional magnetic field $B_{\mathrm{N}} = A_{\mathrm{H}} \langle I_z \rangle / (g^{*} \mu_B)$, where g^{*} is the effective electron g–factor and μ_B is the Bohr magneton. This so-called Overhauser field modifies the electronic Zeeman energy $E_z \propto (B + B_{\mathrm{N}})$. Note that the negative value of the g–factor of GaAs causes a negative B_{N}-field [5], eventually reducing E_z. This decreases the separation between LL0↑ and LL0↓, and therefore, it enhances the inter-LL scattering causing the non-zero longitudinal resistance in the QH breakdown regime.

Likewise, polarized electron spins act on the nuclear spins by adding the local magnetic field $B_{\mathrm{e}} = b_0 \langle S_z \rangle$ (b_0: constant). Accordingly, the NR frequency is shifted from the reference NR line position in the absence of B_{e}. This shift is, in this study, defined as the Knight shift $K_{\mathrm{s}} = -\gamma_{\mathrm{n}} B_{\mathrm{e}}/2\pi$, where γ_n is the nuclear gyromagnetic factor.

Supplementary Note 4. V_x-I_x curve

Supplementary Figure 2 displays a V_x-I_x curve measured at ν =1.05 and $B = 8$ T. V_x vanishes near $I_x = 0$ μA owing to the dissipation-less integer QH; however, it increases abruptly above $I_x = 0.5$ μA, indicating the breakdown of the QH effect. The point marked by the cross indicates the measurement conditions for Fig. 1c.

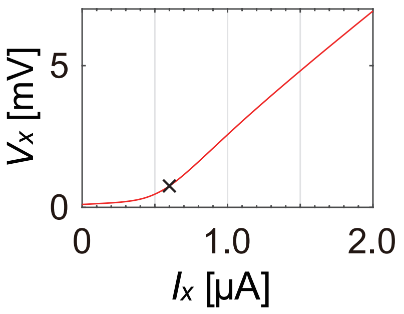

Supplementary Figure 2: V_x-I_x **curve.** V_x-I_x curve was measured at ν =1.05 and B = 8 T. The cross marks the measurement conditions for Fig. 1c.

Supplementary Note 5. Local steady-state NR measurement

The low-field tail below 115.7 MHz in the NR spectrum (Fig. 1c) is an artefact resulting from fast sweeping from high to low frequencies. To measure the exact shape of the NR spectrum, we performed steady-state measurements near position i (see inset of Fig. 1c) at experimental conditions used in the measurement in Fig. 1c. After pumping the DNP at the off-resonance frequency $f_{\rm off}$ = 115.90 MHz for $t_{\rm off}$ > 15 s (Supplementary Note 6), we performed NR at the on-resonance frequency for $t_{\rm on}$ = 12–15 s until the steady state was attained (Supplementary Note 6) and we eventually measured the variation in V_x (ΔV_x). By repeating these procedures in the range of $f_{\rm on}$ = 115.67–115.83 MHz, the steady-state NR spectrum was obtained. During the whole sequence, all other conditions of I_x, ν, and B were maintained constant. As shown in Supplementary Fig. 3, the resulting spectrum shows the NR peak near f = 115.73 MHz with asymmetric broadening in the region approximately between 115.7 and 115.8 MHz (grey area marked in Fig. 1c).

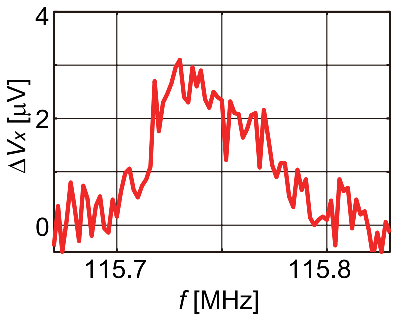

Supplementary Figure 3: **Local steady-state NR spectrum.** The spectrum was taken near the position i (see inset of Fig. 1c) at ν = 1.05, B = 8 T, and I_x = 0.6 μA (marked in the $V_x - I_x$ curve shown in Supplementary Fig. 2).

Supplementary Note 6. Time evolution of V_x

To determine the time duration for the steady-state NR and DNP, we measured the time evolution of V_x by applying the E-field at a point where the DNP arises in the interior of the Hall bar at $\nu \sim 1.0$. Supplementary Figure 4 shows the representative time (t) dependencies of V_x recorded at the same point with different time sequences of switching of the RF E-field frequency. After switching the E-field frequency from the off-resonance to on-resonance at $t = 0$ s, the V_x (bottom curve) started decreasing owing to the NR-induced nuclear spin depolarisation and then saturated at the NR saturation time (typically $t_{\mathrm{NR}} \sim 12$ s). Subsequently, switching back to the off-resonance at $t = 18$ s recovered the V_x, and hence, the DNP. A longer recovery sequence (top curve) demonstrated that the DNP was fully recovered for the recovery time (typically $t_{\mathrm{DNP}} \sim 15$ s). We therefore chose t_{on} =12–15 s for reaching the steady-state NR, and $t_{\mathrm{off}} > 15$ s for initialising the DNP.

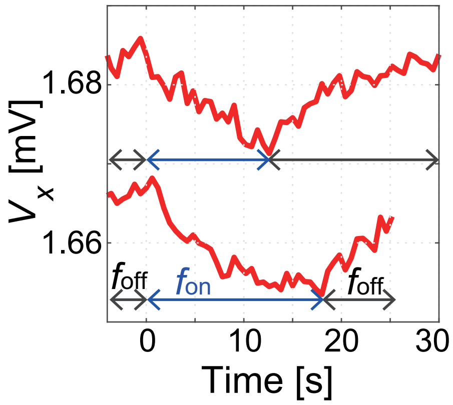

Supplementary Figure 4: **Time evolution of V_x for NR saturation and DNP recovery.** The top and bottom time dependencies of V_x were recorded with different time sequences for the switching of on (f_{on}) and off (f_{off}) resonance frequencies at a point where the maximum DNP arises in the interior of the Hall bar at $\nu \sim 1.0$ and $B = 7$ T for sample A. The switching sequences of E-field between on- and off-resonance frequencies are marked by arrows.

Supplementary Note 7. Determination of NR frequencies at the maximum and zero Knight shift

To determine $2f_{^{75}\mathrm{As}}$ at the maximum and zero Knight shift as a reference, we performed global pump-and-probe measurements at the fully spin-polarised $\nu = 1$ and spin unpolarised $\nu = 2$ QH states, respectively. We first pumped DNP in the QH breakdown regime at $I_x = 1.7$ μA and

ν = 1.05 for t = 77 s. Subsequently, once the current had been turned off, the frequency of the RF E-field generated by the back gate was set to arbitrary $f_{\rm on}$ in the range from 115.70 to 115.82 MHz at $P_{\rm RF}$ = -8 dBm and maintained for $t_{\rm on}$ = 6 s for NR. Finally, the frequency was switched to $f_{\rm off}$=115.90 MHz with both ν and the current switched back to the QH breakdown regime. The variation in V_x (ΔV_x) caused by NR was then probed. By repeating the pump-and-probe measurements with sweeping $f_{\rm on}$, an NR spectrum was obtained. As shown in Supplementary Fig. 5, the spectrum (red dots) obtained at ν = 2 was fitted by the function (Methods) at K_s = 0 kHz to determine $2f_{^{75}\rm As}$ = 115.8090 kHz and the spectrum-line width Γ = 4.63 kHz. Then, the spectrum obtained at ν = 1 (black dots) was fitted using the Γ and $2f_{^{75}\rm As}$ without the Knight shift to determine $2f_{^{75}\rm As}$ at the maximum Knight shift, $2f_{^{75}\rm As}$ = 115.7216 MHz. As a result, the maximum Knight shift was determined as $K_{\rm s}$ = 43.7 kHz. Note that we used half the value of $2K_{\rm s}$.

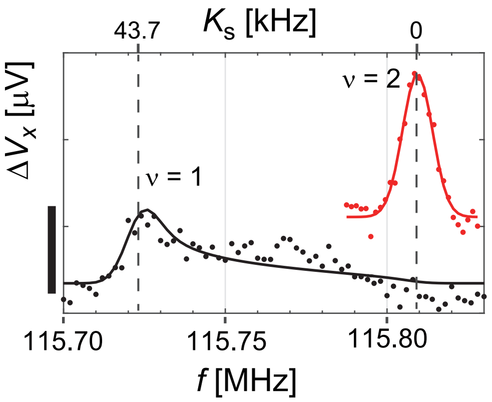

Supplementary Figure 5: **Global NR spectra measured at the fully spin-polarised ν = 1 and the spin-unpolarised ν = 2 QH states.** The spectra (dots) were captured at B = 8 T for sample B and then fitted by the function (Methods). The curves are vertically offset for clarity; the vertical scale bar represents 5 μV.

Supplementary Note 8. $I_x - \nu$ plots of V_x

Supplementary Figure 6 displays the colour-scale $I_x - \nu$ plots of V_x measured for sample A at B = 7 T. The dissipation-less integer QH state represented by $V_x \simeq 0$ (black area) breaks down with an abrupt increase in V_x above the critical current $I_{\rm c}$ (near the boundary of black and red). Points marked by white crosses indicate the measurement conditions for Fig. 2c-e.

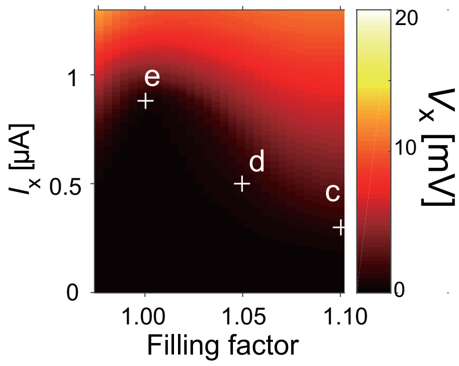

Supplementary Figure 6: **Colour-scale $I_x - \nu$ plots of V_x showing abrupt increment owing to QHE breakdown around $\nu = 1$ at B = 7 T for sample A.**

Supplementary Note 9. RF power dependence of the NR intensity

Supplementary Figure 7 shows the typical RF power dependence of the NR intensity that was extracted from the NR-intensity mappings measured at the centre of the Hall bar in the $\nu \sim 1.0$ QH breakdown regime. To obtain NR intensity sufficient for imaging, we used relatively large RF power $P_{\mathrm{RF}} = 3$ dBm ($\sim$ 2.0 mW), which is in the nonlinear response regime. The observed trend of the ν-dependent distribution in the NR intensity is also confirmed at lower RF power P_{RF} = -10 dBm (0.1 mW) as shown in Fig. 1c. Compared with the NR intensity mapping, relatively smaller RF power [$P_{\mathrm{RF}} = -4$ dBm ($\sim$ 0.4 mW)] was used in the spectroscopy mapping.

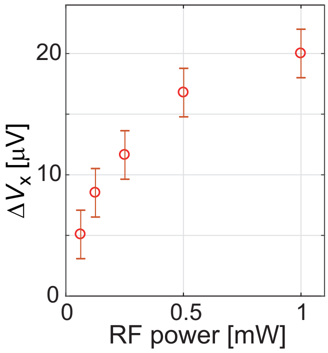

Supplementary Figure 7: **The RF power dependence of the NR intensity ΔV_x.** The ΔV_x [replotted with a different unit (x axis) from Ref [6] ; used in accordance with the Creative Commons Attribution (CC BY) license] represents the maximum ΔV_x measured at the center of the Hall bar in the $\nu \simeq 1.0$ QH breakdown regime at $B = 7$ T after subtracting the background, i.e.ΔV_x observed outside the Hall bar. The error bar represents the fluctuation level estimated from the spatial fluctuation of the back ground.

Supplementary Note 10. Dependence of NR intensity patterns on electron-drift direction

The edge patterns observed at ν=1.10 (Supplementary Fig. 8) appeared along the higher μ_{chem} mesa edge, which was determined as the left (right) side when electrons drift in the direction indicated by the down (up) arrow; H and L are marked as the high and low μ_{chem}-edge sides, respectively.

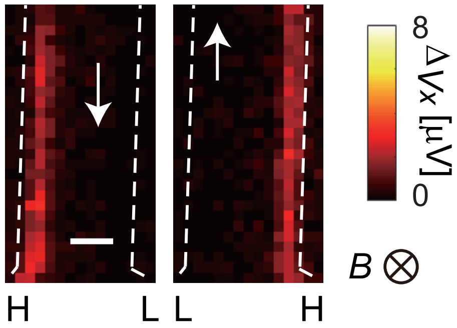

Supplementary Figure 8: **Dependence of NR intensity patterns on direction of electron drift.** The edge pattern in the NR intensity images taken at $\nu = 1.10$ at opposite electron drift directions indicated by arrows: $I_x = 0.70$ μA (left) and -0.71 μA (right). The notations H and L mark the higher and lower μ_{chem} mesa edges, respectively. The scale bar represents 4 μm.

Supplementary Note 11. NR intensity mapping at fixed RD sensitivity.

The incompressible region where LL scattering dominantly occurs can affect local resistive-detection (RD) sensitivity. We tested the NR intensity mapping at fixed RD sensitivity by using the pump–and–probe technique: using the back gate, the filling factors for pumping DNP ($\nu_{\rm pump}$) were set to arbitrary values while that for probing NR intensity ($\nu_{\rm probe}$) was fixed. For NR intensity mapping (see Methods), ν was set to $\nu_{\rm pump}$, and a fixed $\nu_{\rm probe}$ of 1.06 during the application of $f_{\rm off}$ and $f_{\rm on}$ was used (see Fig. 2a). This pump-and-probe-based NR intensity mapping was conducted along a line across the Hall bar. The resulting $\nu_{\rm pump}$-dependence of the NR intensity curves (Supplementary Fig. 9) reproduced the ν-dependent transition of the pattern from the edge (at $\nu_{\rm pump} = 1.13$) to the bulk (at $\nu_{\rm pump} = 1.00$). Thus, we conclude that the ν-dependent patterns shown in Fig. 2(c)–(e) indicate the spatial distributions of the DNP pumped at each ν.

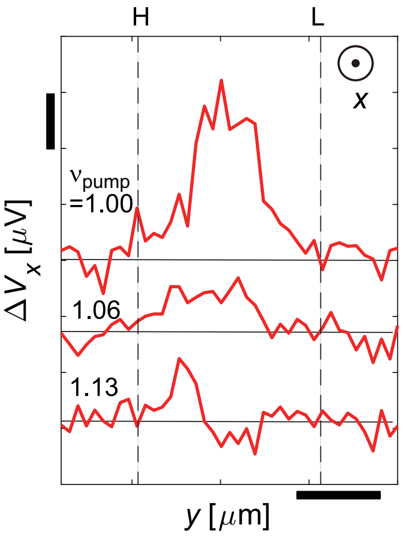

Supplementary Figure 9: **Pump-and-probe-based NR intensity mapping.** NR intensity curves obtained at arbitrary $\nu_{\rm pump}$ and fixed $\nu_{\rm probe}$ at $B = 6.5$ T along a line across the Hall bar (sample B). For NR intensity mapping (see Methods), I_x and ν were set to $I_{x,\rm pump}= 0.8$ μA and $\nu_{\rm pump}$ (as indicated in the figure) while applying the RF at $f_{\rm off} = 94.2$ MHz at $P_{\rm RF} = -4$ dBm for $t_{\rm off} > 15$ s. They were switched to $I_{x,\rm probe}= 0.4$ μA and $\nu_{\rm probe}= 1.06$ during the application of the RF at $f_{\rm on} = 94.03$ MHz with $f_{\rm mod} = \pm 30$ kHz for $t_{\rm on} = 12$–15 s (Supplementary Note 6), and the reduction (ΔV_x) in the V_x was measured. The curves are vertically offset for clarity. The broken vertical lines indicate the mesa-edge positions of the Hall bar with notations for higher (H) and lower (L) $\mu_{\rm chem}$ mesa edges, which were determined by the directions of electron drift (x direction as indicated in the figure) and the B–field (up to down). The solid horizontal lines indicate the zero level of NR intensity. The vertical and horizontal scale bars represent 1 μV and 5 μm, respectively.

Supplementary Note 12. NR spectroscopic mapping

NR-spectroscopic data was constructed by mapping ΔV_x at arbitrary frequencies corresponding to f_{on} in the range 115.730 MHz–115.770 MHz at 50-kHz steps. ΔV_x was mapped by using the same procedure as shown in Fig. 2a. After positioning the tip to a measuring point, the RF E–field was set to f_{on} and $P_{\mathrm{RF}} = -4$ dBm for $t_{\mathrm{on}} = 12$–13 until the steady state was attained (Supplementary Note 6). The frequency was then switched back to $f_{\mathrm{off}} = 115.95$ MHz, and an NR-induced reduction of V_x (ΔV_x) was measured, following which V_x was recovered and the tip moved to the next position for a time interval higher than the DNP recovery time of 15 s (Supplementary Note 6). Supplementary Figure 10a shows the representative ΔV_x images taken at arbitrary f_{on} as indicated above each image and marked by arrows on the global spectrum (Supplementary Fig. 10b). We extracted intensity at fixed spatial points from the dataset of the ΔV_x images for different values of f_{on} to reconstruct the local NR spectra shown in Fig. 3e.

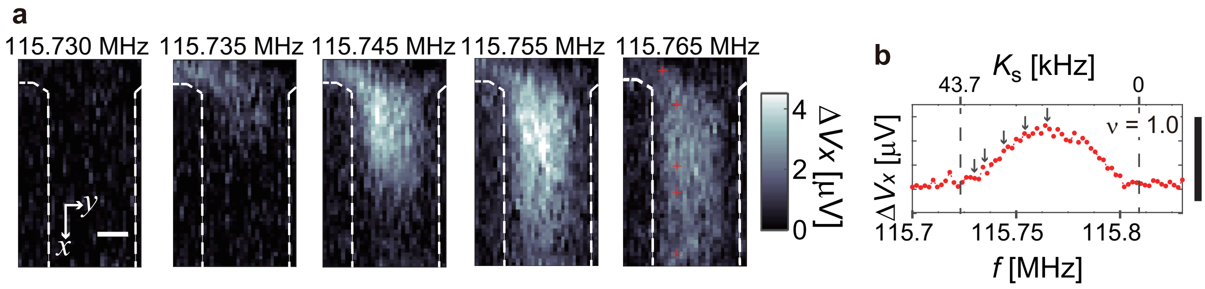

Supplementary Figure 10: ΔV_x **images taken in the area marked by the broken square in Fig. 3b at fixed** f_{on} **and** $I_x = 2.6$ μ**A.** The measurement f_{on} indicated above each image (a) is marked by arrows on the global spectrum (b: the same NR spectrum as shown in Fig. 3a). The scale bars in (a) and (b) represent 4 μm and 10 μV, respectively.

Supplementary Note 13. Comparison of global NR spectra measured at different voltage probes of the Hall bar

Compared to the NR spectra captured at V_{x23} (Supplementary Fig. 11c; the same as Fig. 3a), the spectra at V_{x12} (Supplementary Fig. 11b) resembles the Knight shift at I_x= 1.7-2.6 μA. This indicates that the electron injected from the upper-side voltage probe 1 (see Supplementary Fig. 11a) did not undergo further depolarisation below the lower-side voltage probe 2, but spin polarisation in the two areas resembled. This suggests that electrons heated up by the avalanche process are cooled down in each voltage probe.

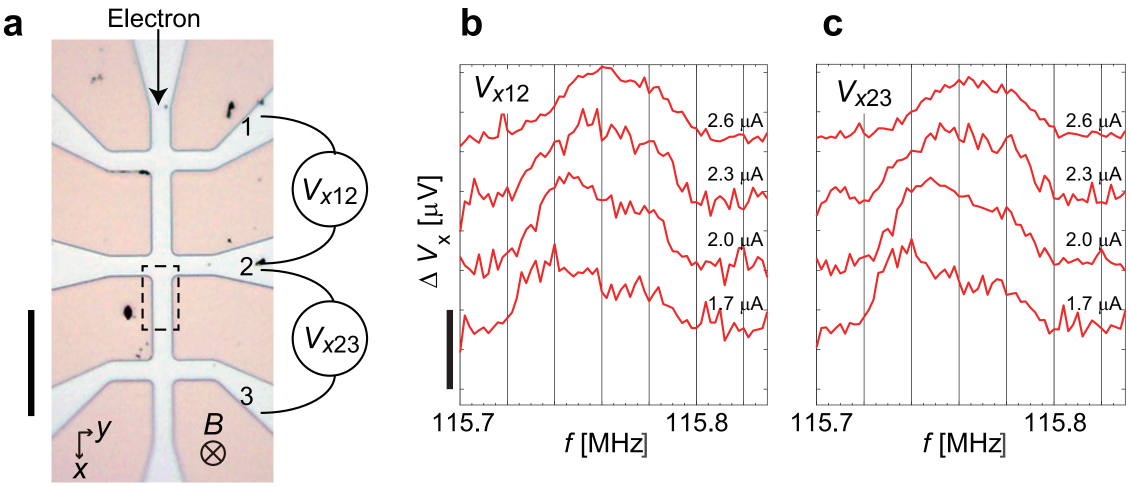

Supplementary Figure 11: **Comparison of global NR spectra measured at different voltage-probe pairs of the Hall bar: sample B. a**. Optical micrograph of the Hall bar with voltage probes 1-3 (vertical scale bar, 50 μm). The broken square corresponds to the measurement area marked in Fig. 3b. **b, c**. Global NR spectra obtained at $\nu = 1.02$ at different currents (indicated in the figures) by measuring V_{x12} (b) and V_{x23} (c) (the same spectra as in Fig. 3a). The curves are vertically offset for clarity. The vertical scale bar represents 10 μV.

Supplementary References

[1] Slichter, C. P. *Principles of magnetic resonance*, vol. 1 (Springer Science & Business Media, 2013).

[2] Bolef, D. *Nuclear acoustic resonance* (Academic Press, 2012).

[3] Brun, E., Mahler, R., Mahon, H. & Pierce, W. Electrically induced nuclear quadrupole spin transitions in a gaas single crystal. *Physical Review* **129**, 1965–1970 (1963).

[4] Kempf, J. G. & Weitekamp, D. P. Method for atomic-layer-resolved measurement of polarization fields by nuclear magnetic resonance. *Journal of Vacuum Science & Technology B: Microelectronics and Nanometer Structures Processing, Measurement, and Phenomena* **18**, 2255–2262 (2000).

[5] Kawamura, M. *et al.* Electrical polarization of nuclear spins in a breakdown regime of quantum Hall effect. *Applied physics letters* **90**, 022102 (2007).

[6] Hashimoto, K. *et al.* Scanning nuclear electric resonance microscopy using quantum-Hall-effect breakdown. *AIP Advances* **6**, 075024 (2016).
